# The Prevalence Rates of Colistin Resistance Among Third-Generation Cephalosporin-Resistant *E. coli* Isolates From Thai Patients

**DOI:** 10.1155/tswj/9921256

**Published:** 2025-10-22

**Authors:** Siriporn Kowaboot, Nipaporn Tewawong, Apichai Sreepian, Preeyaporn M. Sreepian, Utsanee Supcharoengoon, Aunchalee Tonsomboon, Naiyana Watanagul, Pannamthip Pitaksajjakul

**Affiliations:** ^1^Faculty of Medical Technology, Rangsit University, Pathum Thani, Thailand; ^2^Department of Microbiology, Nopparat Rajathanee Hospital, Bangkok, Thailand; ^3^Department of Social and Environmental Medicine, Faculty of Tropical Medicine, Mahidol University, Bangkok, Thailand; ^4^Center of Excellence for Antibody Research, Faculty of Tropical Medicine, Mahidol University, Bangkok, Thailand

**Keywords:** colistin resistance, *E. coli*, *mcr-1*, *mcr-3*

## Abstract

Drug-resistant infections, such as colistin resistance, are reportedly increasing due to the inappropriate use of antibiotics, lack of good control, and the use of excessive quantities of antibiotics. Colistin resistance has been observed in bacteria of the order *Enterobacterales* exhibiting resistance to third-generation cephalosporins or carbapenems. Currently, the prevalence of colistin resistance in third-generation cephalosporin-resistant *E. coli* obtained from patients at Nopparat Rajathanee Hospital, Thailand, in 2023 was determined by broth microdilution. Among 343 isolates, 1.45% (95% CI: 0.19%–2.73%, *n* = 5/343) exhibited a minimum inhibitory concentration (MIC) of colistin between 4 and 8 *μ*g/mL, indicating colistin-resistant *E. coli*. Nevertheless, five isolates were positive for resistant genes by multiplex PCR: two isolates for *mcr*-1, two isolates for *mcr*-3, and one isolate for both *mcr*-1 and *mcr*-3. This study reveals crucial data on resistance genes, informing surveillance of antibiotic resistance, treatment decisions, and public health initiatives to combat resistant bacteria.

## 1. Introduction

Globally, the escalating number of infections arising from antimicrobial-resistant organisms presents a major public health challenge. One primary driver of this trend is the misuse and overuse of antimicrobial agents, often due to poor infection control and the unnecessary prescription of antimicrobial drugs. These factors have accelerated the spread of resistance, limiting the effectiveness of available treatments and allowing resistant bacteria to persist in the environment. In Thailand, antimicrobials are commonly applied in healthcare settings, agriculture, and animal production systems. Gram-negative bacteria, especially members of the *Enterobacterales* order, such as *Escherichia coli* (*E. coli*), are frequently responsible for hospital-acquired infections. These organisms frequently demonstrate resistance to broad-spectrum antibiotics, encompassing *β*-lactams, aminoglycosides, fluoroquinolones, and carbapenems. The rise of multidrug-resistant strains has led to colistin's reintroduction as a treatment option [1].

Colistin (Polymyxin E) is a potent antibiotic that is effective against gram-negative bacteria, including *Enterobacterales* and nonfermentative species [2]. The bactericidal effect of colistin, a polymyxin, involves binding to the lipopolysaccharide of gram-negative bacteria. The interaction of the positively charged polypeptide of colistin with the membrane compromises its integrity, ultimately causing cell lysis. As the use of colistin has increased, the occurrence of colistin resistance among gram-negative bacteria has also increased globally, with resistance rates steadily increasing [3].

In 2015, the discovery of the *mcr-1* gene, representing the first known instance of a plasmid-mediated colistin resistance gene in colistin-resistant *E. coli*, which was isolated from Chinese pig meat, marked a significant advancement [4]. Since this initial discovery, a series of related gene variants, designated *mcr-1* through *mcr-10*, have been identified on plasmids in colistin-resistant *Enterobacterales* strains [5–10]. Among the *mcr* genes, *mcr-1* is the most frequently observed, especially in environments related to farm animals and in patients with *Enterobacterales* infections [11]. In Thailand, the initial identification of *mcr-1* occurred in *E. coli* isolates exhibiting resistance to colistin. In addition, all of them also presented resistance to third-generation cephalosporins [12]. Recently, the *mcr-3* gene has been increasingly identified in *E. coli* strains derived from human clinical samples [13]. Therefore, the objective of this study was to ascertain the prevalence of both *mcr-1* and *mcr-3* genes exhibiting colistin resistance in third-generation cephalosporin-resistant *E. coli* isolated from patient samples in Thailand between March and September 2023. The findings of this study will provide the essential data to elucidate the prevalence of resistance genes, thereby enabling the surveillance of antibiotic resistance patterns, guiding therapeutic strategies, and supporting the development of effective public health measures to control the dissemination of antibiotic-resistant bacteria.

## 2. Materials and Methods

### 2.1. Sample Collection and Identification

Previous microbiological analyses resulted in the successful isolation and identification of 343 *E. coli* via matrix-assisted laser desorption ionization time-of-flight mass spectrometry (MALDI-TOF MS) at Nopparat Rajathanee Hospital, Thailand. The isolates were obtained from patients from March to September 2023. The confirmation of all isolates as *E. coli* was performed by 16S rRNA PCR amplification [14].

### 2.2. Antimicrobial Susceptibility Testing

Antimicrobial susceptibility testing of *E. coli* was done by the Sensititre ARIS 2X (Thermo Fisher Scientific, United Kingdom) as part of routine microbiology, according to the guidelines of Clinical and Laboratory Standards Institute (CLSI) [15]. The antibiotics evaluated in this study were ampicillin (AMP), amoxicillin–clavulanate (AMC), cefoxitin (FOX), cefotaxime (CTX), ceftriaxone (CRO), ceftazidime (CAZ), imipenem (IMP), meropenem (MEM), ertapenem (ETP), gentamicin (CN), amikacin (AK), netilmicin (NET), ciprofloxacin (CIP), levofloxacin (LEV), and trimethoprim/sulfamethoxazole (SXT). Colistin resistance was manually determined by broth microdilution. Colistin dilutions ranging from 0.25 to 128 *μ*g/mL were prepared in cation-adjusted Mueller–Hinton Broth (Oxoid, United Kingdom). The results were subsequently analyzed in accordance with the guidelines of the European Committee on Antimicrobial Susceptibility Testing [16]. The isolates exhibiting MICs of 4 *μ*g/mL or greater were categorized as colistin-resistant *E. coli*. This study used *E. coli* ATCC 25922 as a reference strain for drug susceptibility testing. Tests were repeated independently three times. Multidrug resistance (MDR) was defined as nonsusceptibility to at least one agent in three or more antimicrobial categories [17].

### 2.3. Detection of Colistin Resistance Genes via Multiplex PCR

In this study, the boiling method was used to extract the genomic DNA of *E. coli* for the PCR assay [18]. The concentrations of DNA were measured using Nano-400A (Allsheng, Hangzhou, China). The colistin resistance genes, including *mcr-1* and *mcr-3*, were identified through multiplex PCR. A 25 *μ*L of PCR reaction mixture was prepared, including 0.4 *μ*M of each primer (Table 1), 100 ng DNA template, and 1× AccuStart II Geltrack PCR Supermix (Quantabio, Beverly, Massachusetts, United States). The amplification steps were conducted as previously described [19]. Briefly, the thermal cycling started with an initial denaturation (94°C for 3 min), followed by 25 cycles (denaturation: 94°C, 30 s; annealing: 58°C, 90 s; and extension: 72°C, 60 s), and a final extension (72°C, 5 min). The visualization of the PCR products was performed using GelDoc Go after separation by 1.5% agarose gel electrophoresis. Control strain of *E. coli* ATCC25922 was used as a negative control.

### 2.4. Nucleotide Sequence Accession Numbers

The 16S rRNA nucleotide sequences of *E. coli* and colistin resistance genes, *mcr-1*and *mcr-3*, are accessible via GenBank accession numbers PV453565, PV449191, and PV469330, respectively.

### 2.5. Data Analysis

The descriptive analysis of the categorical variables, along with their 95% confidence intervals (CIs), was determined using SPSS Version 21.0 (IBM, United States).

### 2.6. Ethical Approval

The current study was approved by the Ethics Review Board of Rangsit University, under the approval number designated as RSUERB 2022/070.

## 3. Results

### 3.1. Isolation and Characterization of *E. coli*

The demographics and characteristics of the patients infected with *E. coli* admitted to Nopparat Rajathanee Hospital between March and September 2023 are displayed in Table 2. The sample population comprised 54.8% (188/343) females and 45.2% (155/343) males. The age group of 25–60 years represented almost the entire population (87.8%; 301/343). Nearly half of the collected samples were catheterized urine (46.4%; 159/343), followed by blood (16.0%; 55/343) and sputum (13.1%; 45/343). The 16S rRNA gene analysis by PCR amplification confirmed that all 343 isolates were *E. coli*.

### 3.2. Antimicrobial Susceptibility Test

Table 3 presents a comprehensive overview of the resistance percentages observed in the antibiotics tested in this study. The results indicated that all bacterial isolates (343 isolates) in this study were resistant to AMP and three cephalosporin antibiotics, including cefazolin (1st gen), CTX (3rd gen), and CRO (3rd gen). The percentage of bacterial resistance to CIP, LEV, SXT, CAZ, and CN was 78.1%, 75.2%, 63.3%, 44.0%, and 41.1%, respectively. Interestingly, among 343 isolates, colistin resistance was detected in five (1.5%) isolates via the broth microdilution method, with MICs ranging from 4 to 8 *μ*g/mL.

### 3.3. Molecular Identification of *mcr*

**Figure 1:**
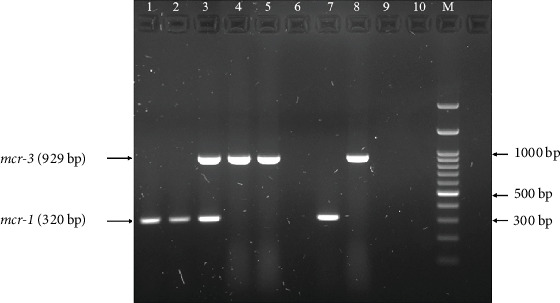
PCR amplification of colistin resistance genes (*mcr*) in colistin-resistant *E. coli*; Lanes 1–5: Isolate Nos. 22, 162, 176, 177, and 182, respectively; Lane 6: negative sample; Lane 7: positive control for the *mcr-1* gene (PV449191); Lane 8: positive control for the *mcr-3* gene (PV469330); Lane 9: negative control (*E. coli* ATCC 25922); Lane 10: negative control (no template); Lane M: DNA ladder 100 bp.

In this study, a multiplex PCR analysis was conducted on 343 isolates of *E. coli* exhibiting cephalosporin resistance to identify *mcr-1* and *mcr-3* genes. The results demonstrated that the *mcr* genes were detected in 1.45% (95% CI: 0.19%–2.73%, *n* = 5/343). In addition, the *mcr* genes were detected in 100% (5/5) of the isolates, which demonstrated colistin resistance (Figure 1). The information regarding *E. coli* isolates that show positive results for *mcr*-1 and *mcr*-3 is shown in Table 4. The positive isolates were collected from various departments. It was observed that most isolates were derived from patients older than 60 years, except for Isolate Number 176. According to the EUCAST guidelines, all *mcr* gene–positive isolates exhibited colistin MIC values ≥4 *μ*g/mL, which is indicative of colistin resistance. Isolate Numbers 22 and 162 were positive for *mcr*-1 from March to May, whereas Isolate Numbers 177 and 182 were positive for *mcr*-3 from August to September. Notably, Isolate Number 176 was positive for both *mcr*-1 and *mcr*-3 in August. The *mcr* genes were undetectable in *E. coli* isolates with colistin MIC lower than 2 *μ*g/mL.

Following identification as *mcr*-positive, the five bacterial strains were assessed for antibacterial susceptibility to various antibiotics. Among these five strains, three strains exhibited MDR, while the remaining two strains demonstrated MDR in addition to carbapenem resistance. All five isolates were resistant to antimicrobial classes with different patterns (Table 5).

## 4. Discussion

Colistin may be reserved as a final treatment option for *E. coli* infections that have developed resistance to multiple antibiotics, including those in the third-generation cephalosporins, which are among the commonly used antibiotics. The rise of third-generation cephalosporin-resistant *E. coli* infection has directly contributed to the faster emergence of colistin resistance. The bacteria used in this study were *E. coli* strains that exhibited resistance to third-generation cephalosporins. Our observations revealed a high resistance rate among *E. coli* to fluoroquinolones, ranging from 75% to 78%, which is higher than that reported previously [20–22]. One to two-thirds of Enterobacteriaceae that produce extended-spectrum *β*-lactamases are resistant to fluoroquinolone [23]. Fluoroquinolone-resistant *E. coli* isolated from a UTI patient in Thailand revealed that the presence of the PMQR gene is related to ESBL strains [24]. Nevertheless, our study revealed that 55 *E. coli* isolates were susceptible to fluoroquinolones (CIP and LEV).

This study revealed a low prevalence of *E. coli* isolates exhibiting resistance to colistin, with 1.46%. A similar result has been reported in previous studies [25–28]. In addition, in our study, the prevalence rate of *E. coli* exhibiting colistin resistance was lower than that reported by Boonyasiri et al. in 2023 in Thailand, who found that the prevalence rates of colistin-resistant *E. coli* in patients, healthy farmers, and pigs were 12.1%, 10.2%, and 68.4%, respectively [13].

In this study, plasmids encoding *mcr-1*, *mcr-3*, or a combination of the two genes were identified in *E. coli* with existing colistin resistance. Two isolates carried a single *mcr-1* gene, two carried a single *mcr-3* gene, and one carried both genes. Several previous studies have indicated that *mcr-1* is more common than other *mcr* genes [26, 29–31]. Furthermore, a significant finding from recent research indicates that the *mcr*-3 gene was detected at a high rate (54%) within colistin-resistant *E. coli* samples obtained from various pig farms in Thailand [19]. The prevalence of the *mcr-1* gene reported by Boonyasiri et al. [13] was 52.9% in colistin-resistant *E. coli* isolated from porcine fecal samples and 87.5% in those from human fecal samples.

In contrast, the *mcr-3* gene was found in 37.5% of human fecal isolates and up to 96.1% of pig fecal isolates. These findings reveal that *mcr-1* is more commonly found in *E. coli* from human fecal samples than in those from porcine fecal samples, whereas *mcr-3* is more common in isolates from porcine fecal samples than in those from human feces. A 2017 study on antibiotic usage revealed that small to medium-sized pig farms in the northeastern region of Thailand were increasingly using amoxicillin and colistin [32]. Colistin is currently banned as a feed additive in Thailand. Nevertheless, the prolonged and cumulative selective pressure from its prior use may have contributed to the development of bacterial resistance to the drug. This effect may explain the occurrence of colistin-resistant *E. coli* carrying the *mcr-3* gene, suggesting the possibility of transmission from animals to humans. The discovery of the *mcr-3* gene underscores the potential for the development of colistin resistance within the healthcare settings in Thailand. In addition, the co-occurrence of two *mcr* plasmids has rarely been reported. However, we found one isolate that carried both *mcr-1* and *mcr-3*. The co-occurrence of the *mcr-1* and *mcr-3* genes within a single isolate of *E. coli* from human patients has also recently been reported in China and New Zealand [33, 34].

Our study revealed that *E. coli* strains displaying either *mcr-1* or *mcr-3* presented a moderate level of colistin resistance, with MICs ranging from 4 to 8 *μ*g/mL. This finding aligns with those of previous studies conducted in China and Nepal [35, 36]. These results suggest that the simultaneous presence of *mcr-1* and *mcr-3* does not increase colistin resistance. One notable difference between the *mcr-1* and *mcr-3* genes is that *mcr-3* has greater heat stability. This fact could explain why, even if the *mcr-1* gene is destroyed, resistance is not eliminated, as the resistance phenotype is maintained through the properties of the *mcr-*3 gene [37]. Our study has several limitations. The current study was limited to *mcr-1* and *mcr-3*. According to other research, *mcr-1* was mostly carried by conserved epidemic plasmids, while *mcr-3* was located on a more diverse set of plasmid types [38]. However, isolation and characterization of *mcr-2* and *mcr-4* to *mcr-10* are suggested in future studies to predict the role of genes in conferring resistance. Our analysis revealed no association between infections caused by *mcr*-1- or *mcr*-3-carrying *E. coli* and the infection site, time period, or specific wards. However, multilocus sequence typing (MLST) or whole-genome sequencing (WGS) would provide a higher level of resolution for species confirmation and for understanding the evolution of these *mcr*-carrying *E. coli* within the hospital. As the present study was conducted in a single hospital, the reported rates correspond to a limited geographical area and may not be directly extrapolated.

## 5. Conclusion

A global distribution of the *mcr* gene has been observed, with surveillance efforts encompassing *E. coli* from human, animal, and environmental samples. The occurrence of colistin resistance and the presence of the *mcr* gene in clinical isolates from our study represent a considerable public health concern in hospitals. Therefore, the prompt implementation of antibiotic stewardship programs is essential for controlling infections.

## Figures and Tables

**Table 1:** Primer sequences and PCR product sizes.

**Target genes**	**Primer sequence**	**bp.**	**Ref.**
*mcr-1*	F-5⁣′-AGTCCGTTTGTTCTTGTGGC-3⁣′ R-5⁣′-AGATCCTTGGTCTCGGCTTG-3⁣′	320	18
*mcr-3*	F-5⁣′-AAATAAAAATTGTTCCGCTTATG-3⁣′ R-5⁣′-AATGGAGATCCCCGTTTTT-3⁣′	929	18
16s rRNA	F-5⁣′-AGAGTTTGATCMTGGCTCAG-3⁣′ R-5⁣′-CCGTCAATTCATTTGAGTTT-3⁣′	919	14

**Table 2:** Demographics and characteristics of patients infected with *E. coli* attending Nopparat Rajathanee Hospital during March to September 2023.

**Character**	**No. of total samples**	**%**
Gender		
Male	155	45.2
Female	188	54.8
Age group (years)		
0–14	7	2.0
15–24	3	0.9
25–60	301	87.8
>60	32	9.3
Clinical samples		
Pus	29	8.5
Fluid	11	3.2
Tissue	12	3.5
Sputum	45	13.1
Urine (catheter)	32	9.3
Catheterized urine	159	46.4
Blood	55	16.0

**Table 3:** Antibiotic susceptibilities of the 343 *E. coli* isolates.

**Antibiotics**	**S** **(%)**	**I** **(%)**	**R** **(%)**
Ampicillin (AMP)	0 (0.00)	0 (0.00)	343 (100.00)
Amoxicillin–clavulanic (AMC)	218 (63.56)	68 (19.83)	57 (16.62)
Cefoxitin (FOX)	259 (75.51)	29 (8.45)	55 (16.03)
Cefotaxime (CTX)	0 (0.00)	0 (0.00)	343 (100.00)
Ceftriaxone (CRO)	0 (0.00)	0 (0.00)	343 (100.00)
Ceftazidime (CAZ)	128 (37.32)	64 (18.66)	151 (44.02)
Imipenem (IMP)	328 (95.63)	3 (0.87)	12 (3.50)
Meropenem (MEM)	327 (95.34)	2 (0.58)	14 (4.08)
Ertapenem (ERT)	321 (93.59)	1 (0.29)	21 (6.12)
Gentamicin (CN)	199 (58.02)	3 (0.87)	141 (41.11)
Amikacin (AK)	340 (99.13)	0 (0.00)	3 (0.87)
Netilmicin (NET)	295 (86.01)	37 (10.79)	11 (3.21)
Ciprofloxacin (CIP)	56 (16.33)	19 (5.54)	268 (78.13)
Levofloxacin (LEV)	66 (19.24)	19 (5.54)	258 (75.22)
Trimethoprim/sulfamethoxazole (SXT)	126 (36.73)	0 (0.00)	217 (63.27)
Colistin (CT)	0 (0.00)	338 (98.54)	5 (1.46)

Abbreviations: *I*, intermediate; *S*, susceptibility; *R*, resistance.

**Table 4:** Information on the five *mcr-1-* or *mcr-3*-harboring *Escherichia coli*.

	**No. of isolates of *E. coli***				
	**22**	**162**	**176**	**177**	**182**
Month of isolation (2023)	March	May	August	August	September
Type of specimen	Sputum	Pus	Pus	Blood	Catheterized urine
Age (years)	63	70	30	86	85
Gender	Male	Female	Male	Male	Female
Ward	Internal medicine	Operating room	Male surgical ward	Male medical ward	Female surgical ward
Colistin MIC (*μ*g/mL)	8	4	8	8	4
*mcr* type	1	1	1, 3	3	3

Abbreviation: MIC, minimum inhibition concentration.

**Table 5:** Minimum inhibitory concentrations for *mcr-1-* or *mcr-3*-harboring *Escherichia coli*.

**Type**	**Antibiotic**	**No. 22 (mcr-1)**		**No. 162 (mcr-1)**		**No. 176 (mcr-1, 3)**		**No. 177 (mcr-3)**		**No. 182 (mcr-3)**	
		**MIC**	**Result**	**MIC**	**Result**	**MIC**	**Result**	**MIC**	**Result**	**MIC**	**Result**
Beta-lactam	AMP	>16	*r*	>16	*r*	>16	*r*	>16	*r*	>16	r

Beta-lactamase inhibitor	AMC	>16/8	*r*	>16/8	*r*	≤4/2	*s*	8/4	*s*	8/4	s

Cephalosporin	FOX	>16	*r*	>16	*r*	≤4	*s*	8	*s*	8	s
	CTX	>32	*r*	>32	*r*	>32	*r*	16	*r*	>32	r
	CRO	>32	*r*	>32	*r*	>32	*r*	32	*r*	>32	r
	CAZ	>32	*r*	32	*r*	8	*i*	≤1	*s*	32	r

Carbapenem	IMP	≤0.5	*s*	1	*s*	≤0.5	*s*	≤0.5	*s*	≤0.5	s
	MEM	≤0.5	*s*	1	*s*	≤0.5	*s*	≤0.5	*s*	≤0.5	s
	ERT	4	*r*	4	*r*	≤0.5	*s*	≤0.5	*s*	≤0.5	s

Aminoglycoside	CN	≤2	*s*	>8	*r*	≤2	*s*	>8	*r*	>8	r
	AK	≤8	*s*	≤8	*s*	≤8	*s*	≤8	*s*	≤8	s
	NET	≤8	*s*	≤8	*s*	≤8	*s*	16	*i*	≤8	s

Fluoroquinolones	CIP	>2	*r*	>2	*r*	>2	*r*	>2	*r*	>2	r
	LEV	>8	*r*	>8	*r*	>8	*r*	>8	*r*	>8	r

Sulfomanide	SXT	≤1/19	*s*	>4/76	*r*	≤1/19	*s*	≤1/19	*s*	≤1/19	s

Polymyxin	CT	8	*r*	4	*r*	8	*r*	8	*r*	4	r

*Note:* The unit for MIC was *μ*g/mL.

Abbreviations: AK, amikacin; AMC, amoxicillin–clavulanate; AMP, ampicillin; CAZ, ceftazidime; CIP, ciprofloxacin; CN, gentamicin; CRO, ceftriaxone; CT, colistin; CTX, cefotaxime; ETP, ertapenem; FOX, cefoxitin; IMP, imipenem; LEV, levofloxacin; MEM, meropenem; NET, netilmicin; SXT, trimethoprim/sulfamethoxazole.

## Data Availability

The data used to support the findings of this study can be obtained from the corresponding author upon reasonable request.

## References

[1] Li J., Nation R. L., Turnidge J. D. (2006). Colistin: The Re-Emerging Antibiotic for Multidrug-Resistant Gram-Negative Bacterial Infections. *Lancet Infectious Diseases*.

[2] Tan T. Y., Ng S. Y. (2006). The In-Vitro Activity of Colistin in Gram-Negative Bacteria. *Singapore Medical Journal*.

[3] Gales A. C., Jones R. N., Sader H. S. (2011). Contemporary Activity of Colistin and Polymyxin B Against a Worldwide Collection of Gram-Negative Pathogens: Results From the SENTRY Antimicrobial Surveillance Program (2006-09). *The Journal of Antimicrobial Chemotherapy*.

[4] Liu Y. Y., Wang Y., Walsh T. R. (2016). Emergence of Plasmid-Mediated Colistin Resistance Mechanism MCR-1 in Animals and Human Beings in China: A Microbiological and Molecular Biological Study. *Lancet Infectious Diseases*.

[5] Xavier B. B., Lammens C., Ruhal R. (2016). Identification of a Novel Plasmid-Mediated Colistin-Resistance Gene, mcr-2, in Escherichia coli, Belgium, June 2016. *Eurosurveillance: European communicable disease bulletin/European Communities. Commission; Communautés européennes. Commission.-Saint-Maurice, 1995, currens*.

[6] Duggett N. A., Randall L. P., Horton R. A. (2018). Molecular Epidemiology of Isolates With Multiple *mcr* Plasmids From a Pig Farm in Great Britain: The Effects of Colistin Withdrawal in the Short and Long Term. *Journal of Antimicrobial Chemotherapy*.

[7] AbuOun M., Stubberfield E. J., Duggett N. A. (2017). *mcr-1* and *mcr-2 (mcr-6.1)* variant genes identified in *Moraxella* species isolated from pigs in Great Britain from 2014 to 2015. *Journal of Antimicrobial Chemotherapy*.

[8] Yang Y. Q., Li Y. X., Lei C. W., Zhang A. Y., Wang H. N. (2018). Novel Plasmid-Mediated Colistin Resistance Gene mcr-7.1 in *Klebsiella pneumoniae*. *Journal of Antimicrobial Chemotherapy*.

[9] Wang X., Wang Y., Zhou Y. (2018). Emergence of a Novel Mobile Colistin Resistance Gene, mcr-8, in NDM-Producing Klebsiella pneumoniae. *Emerging Microbes & Infections*.

[10] Carroll L. M., Gaballa A., Guldimann C., Sullivan G., Henderson L. O., Wiedmann M. (2019). Identification of Novel Mobilized Colistin Resistance Gene mcr-9 in a Multidrug-Resistant, Colistin-Susceptible Salmonella enterica Serotype Typhimurium Isolate. *MBio*.

[11] Skov R. L., Monnet D. L. (2016). Plasmid-Mediated Colistin Resistance (mcr-1 Gene): Three Months Later, the Story Unfolds. *Euro Surveillance*.

[12] Wongut-sa P., Khumdee P., Samosornsuk W., Yansombat J., Samosornsuk S. (2020). Prevalence of Colistin Resistance Among Third Generation Cephalosporins- or Carbapenem-Resistant Enterobacteriaceae Isolated From Clinical Specimens in Ratchaburi Hospital, Thailand. *Journal of the Medical Technologist Association of Thailand*.

[13] Boonyasiri A., Brinkac L. M., Jauneikaite E. (2023). Characteristics and Genomic Epidemiology of Colistin-Resistant Enterobacterales From Farmers, Swine, and Hospitalized Patients in Thailand, 2014-2017. *BMC Infectious Diseases*.

[14] Momtaz H., Karimian A., Madani M. (2013). Uropathogenic Escherichia coli in Iran: Serogroup Distributions, Virulence Factors and Antimicrobial Resistance Properties. *Annals of Clinical Microbiology and Antimicrobials*.

[16] Satlin M. J., Lewis J. S., Weinstein M. P. (2020). Clinical and Laboratory Standards Institute and European Committee on Antimicrobial Susceptibility Testing Position Statements on Polymyxin B and Colistin Clinical Breakpoints. *Clinical Infectious Diseases*.

[17] Magiorakos A. P., Srinivasan A., Carey R. B. (2012). Multidrug-Resistant, Extensively Drug-Resistant and Pandrug-Resistant Bacteria: An International Expert Proposal for Interim Standard Definitions for Acquired Resistance. *Clinical Microbiology and Infection*.

[18] Tewawong N., Kowaboot S., Kengkarn S., Thawornwan U., Ramasoota P., Suthienkul O. (2024). Characterization of Vibrio parahaemolyticus Isolated From Clinical Specimens and Oysters in Thailand. *Journal of Infection in Developing Countries*.

[19] Nguyet L. T. Y., Keeratikunakorn K., Kaeoket K., Ngamwongsatit N. (2022). Antibiotic Resistant Escherichia coli From Diarrheic Piglets From Pig Farms in Thailand That Harbor Colistin-Resistant mcr Genes. *Scientific Reports*.

[20] Karki D., Dhungel B., Bhandari S. (2021). Antibiotic Resistance and Detection of Plasmid Mediated Colistin Resistance mcr-1 Gene among *Escherichia coli* and *Klebsiella pneumoniae* Isolated From Clinical Samples. *Gut Pathogens*.

[21] Nepal K., Pant N. D., Neupane B. (2017). Extended Spectrum Beta-Lactamase and Metallo Beta-Lactamase Production Among Escherichia coli and Klebsiella pneumoniae Isolated From Different Clinical Samples in a Tertiary Care Hospital in Kathmandu, Nepal. *Annals of Clinical Microbiology and Antimicrobials*.

[22] Yadav M., Khumanthem S., Kshtrimayum M. Antibiotic Resistance Trends of Uropathogenic Escherichia coli Isolated From Inpatients in a Tertiary Care Hospital in North East India. http://www.recentscientific.com/sites/default/files/8080-A-2017.pdf.

[23] Dalhoff A. (2012). Global Fluoroquinolone Resistance Epidemiology and Implictions for Clinical Use. *Interdisciplinary Perspectives on Infectious Diseases*.

[24] Tewawong N., Kowaboot S., Lektrakul W., Supcharoengoon U., Watanagul N., Pitaksajjakul P. (2025). Mechanisms of Fluoroquinolone Resistance Among Escherichia coli Isolates From Urinary Tract Infections in Thailand. *PLoS One*.

[25] Castanheira M., Griffin M. A., Deshpande L. M., Mendes R. E., Jones R. N., Flamm R. K. (2016). Detection of mcr-1 Among *Escherichia coli* Clinical Isolates Collected Worldwide as Part of the SENTRY Antimicrobial Surveillance Program in 2014 and 2015. *Antimicrobial Agents and Chemotherapy*.

[26] Liassine N., Assouvie L., Descombes M. C. (2016). Very Low Prevalence of MCR-1/MCR-2 Plasmid-Mediated Colistin Resistance in Urinary Tract *Enterobacteriaceae* in Switzerland. *International Journal of Infectious Diseases*.

[27] Prim N., Rivera A., Rodríguez-Navarro J. (2016). Detection of mcr-1 Colistin Resistance Gene in Polyclonal *Escherichia coli* Isolates in Barcelona, Spain, 2012 to 2015. *Eurosurveillance*.

[28] Bradford P. A., Kazmierczak K. M., Biedenbach D. J., Wise M. G., Hackel M., Sahm D. F. (2015). Correlation of *β*-Lactamase Production and Colistin Resistance among Enterobacteriaceae Isolates from a Global Surveillance Program. *Antimicrobial Agents and Chemotherapy*.

[29] Al Momani W. M., Ata N., Maslat A. O. (2024). Colistin-Resistance Genes in Escherichia coli Isolated From Patients with Urinary Tract Infections. *PLoS One*.

[30] San N., Aung M. S., Thu P. P. (2019). First Detection of the mcr-1 Colistin Resistance Gene in Escherichia coli From a Patient With Urinary Tract Infection in Myanmar. *New Microbes New Infections*.

[31] Anan M. M. G., El-Seidi E. A., Mostafa M. S., Rashed L. A., El-Wakil D. M. (2021). Detection of Plasmid-Mediated Mobile Colistin Resistance Gene (mcr-1) in *Enterobacterales* Isolates From a University Hospital. *Infection and Drug Resistance*.

[32] Ström G., Halje M., Karlsson D. (2017). Antimicrobial Use and Antimicrobial Susceptibility in Escherichia coli on Small- and Medium-Scale Pig Farms in North-Eastern Thailand. *Antimicrobial Resistance and Infection Control*.

[33] Liu L., Feng Y., Zhang X., McNally A., Zong Z. (2017). New Variant of mcr-3 in an Extensively Drug-Resistant Escherichia coli Clinical Isolate Carrying mcr-1 and blaNDM-5. *Antimicrobial Agents and Chemotherapy*.

[34] Creighton J., Anderson T., Howard J., Dyet K., Ren X., Freeman J. (2019). Co-Occurrence of mcr-1 and mcr-3 Genes in a Single Escherichia coli in New Zealand. *Journal of Antimicrobial Chemotherapy*.

[35] Quan J., Li X., Chen Y. (2017). Prevalence of mcr-1 in *Escherichia coli* and *Klebsiella pneumoniae* Recovered From Bloodstream Infections in China: A Multicentre Longitudinal Study. *Lancet Infectious Diseases*.

[36] Walkty A., Karlowsky J. A., Adam H. J. (2016). Frequency of MCR-1-Mediated Colistin Resistance Among Escherichia coli Clinical Isolates Obtained From Patients in Canadian Hospitals (CANWARD 2008-2015). *CMAJ Open*.

[37] Li H., Yang L., Liu Z. (2018). Molecular Insights Into Functional Differences Between mcr-3- and mcr-1-Mediated Colistin Resistance. *Antimicrobial Agents and Chemotherapy*.

[38] Stosic M. S., Hickman R. A., Lunha K. (2023). Exploring the Epidemiology of mcr Genes, Genetic Context and Plasmids in Enterobacteriaceae Originating From Pigs and Humans on Farms in Thailand. *Journal of Antimicrobial Chemotherapy*.

